# Ketamine in the effective management of chronic pain, depression, and posttraumatic stress disorder for Veterans: A meta-analysis and systematic review

**DOI:** 10.3389/fpsyt.2024.1338581

**Published:** 2024-06-24

**Authors:** Jenny J.W. Liu, Natalie Ein, Julia Gervasio, Clara Baker, Rachel Plouffe, Sonya Wanklyn, Amer M. Burhan, Brenda Lau, Emmanuel Abreu, Thomas Wasiuta, Anthony Nazarov, J. Don Richardson

**Affiliations:** ^1^ MacDonald Franklin Operational Stress Injury Research Centre, London, ON, Canada; ^2^ Department of Psychiatry, Schulich School of Medicine and Dentistry, Western University, London, ON, Canada; ^3^ Division of Psychology, School of Humanities, Social Sciences and Law, University of Dundee, Dundee, United Kingdom; ^4^ Operational Stress Injury Clinic, St Joseph’s Health Care, London, ON, Canada; ^5^ Ontario Shores Centre for Mental Health Sciences, Whitby, ON, Canada; ^6^ Temerty Faculty of Medicine, University of Toronto, Toronto, ON, Canada; ^7^ Department of Anesthesiology, Pharmacology and Therapeutics, Faculty of Medicine, University of British Columbia, Vancouver, BC, Canada; ^8^ Change Pain Clinic, Vancouver, BC, Canada; ^9^ Department of Psychiatry and Behavioural Neurosciences, Faculty of Health Sciences, McMaster University, Hamilton, ON, Canada

**Keywords:** ketamine, military, veteran, mental health, PTSD, depression, pain

## Abstract

**Introduction:**

Ketamine has emerged as a promising treatment alternative for the management of chronic pain. Despite encouraging findings in civilian populations, and favourable results from trials examining its efficacy in military populations, there is still a dearth of information pointing to optimal specifications related to ketamine administration for pain, depression, and posttraumatic stress disorder (PTSD) in military populations. This meta-analysis and systematic review synthesised available evidence on the effectiveness, tolerability, and feasibility of ketamine in the management of chronic pain and mental health conditions in military populations.

**Methods:**

This review followed the Cochrane’s Guide for systematic reviews of interventions and Preferred Reporting Items for Systematic Reviews and Meta-Analyses (PRISMA) as frameworks for data collection and synthesis.

**Results:**

A total of 11 studies and 22 independent samples were retained for data analyses. Across samples, improvements in pain, depression, and PTSD outcomes were evident, with the use of ketamine leading to significant reductions, *g* = 1.76, *SE* = 0.19, 95% *CI* (1.39, 2.13), *Z* = 9.26, *p* <.001. These effect sizes were robust with moderate-to-large effects. In addition, the reductions in symptoms were observed in both active-duty and Veteran groups, and for different routes of ketamine administration, frequencies of ketamine administration, duration of ketamine treatments, dosage, study design, and allowance for concurrent treatments.

**Discussion:**

This review provides a preliminary synthesis of available evidence which suggests that ketamine may be a potential option for the treatment of depression, PTSD, and chronic pain in military populations. The viability of ketamine as an alternative treatment may be particularly impactful for those who are treatment resistant, experience chronic symptoms, and/or have exhausted conventional treatments. More research is warranted in order verify the findings presented in this review.

## Introduction

Ketamine has emerged as a promising alternative treatment for the management of chronic pain and certain mental health conditions. Recent reviews have found evidence for the effectiveness of ketamine in perioperative pain reduction (e.g., 1). Research has also shown that ketamine can be an effective intervention for treatment-resistant posttraumatic stress disorder (PTSD; 2) and major depressive disorder (MDD; 3). Despite promising findings in civilian populations, and promising trials examining its efficacy in military populations [National Library of Medicine (NLM), NCT03088384] (4), there remains a dearth of information available to delineate optimal terms of ketamine administration. These include variabilities in the routes of administration, duration of ketamine treatments, dosage, and population groups targeted. Indeed, a scarce amount of training for interventional practitioners exists for the administration of ketamine, despite a growing interest (5). These heterogeneities underscore the lack of clear consensus in the standards of use for different populations, pain conditions, and in different contexts (1, 6).

As a group, Veterans experience higher rates of chronic pain (7) and differences in underlying pain conditions, mechanisms, and responses relative to the general population (8–10). Both mental health and chronic pain conditions are costly to the individual and to society, limiting employment and increasing reliance on existing healthcare services (11). For Veterans specifically, care seeking often reflects lived experiences that intermingle pain, depression, and PTSD. Meanwhile, the applicability and effectiveness of ketamine to treat physical pain and mental health issues among Veterans has not been adequately elucidated. This review thus hopes to uncover effects that can serve to propel further inquiry and deeper understanding of these multifaceted challenges and nuanced considerations in treatment for pain and mental health comorbidities in Veterans.

The aim of this research is to conduct a meta-analysis and systematic review and synthesise evidence on the effectiveness, tolerability, and feasibility of ketamine in the management of chronic pain depression, and PTSD conditions in military populations. Specifically, we sought to determine: (1) whether ketamine is an effective agent in the management of chronic pain, depression, and/or PTSD; and (2) the relative effectiveness and benefits of ketamine treatments influenced by mode of delivery characteristics (i.e., routes of administration, frequency of administration, duration of ketamine therapy, dose and dose ranges), study characteristics [i.e., sample size, study design (e.g., randomised control trial; RCT), study protocol, concurrent treatment, washout period included, adverse effects considered], and intersecting characteristics of the military populations [i.e., population targeted (active duty or Veteran), treatment resistant, chronicity of conditions, age, citizenship, gender].

## Methods

This review followed the Cochrane’s Guide for systematic reviews of interventions (12) and Preferred Reporting Items for Systematic Reviews and Meta-Analyses (PRISMA; 13) as frameworks for data collection and synthesis. The guidelines included: a search strategy across multiple databases, two levels of screening (title and abstract and full text) against inclusion and exclusion criteria, resolving conflicts at each level, data extraction, data analyses, and data synthesis. For this review, SWIFT-Active Screener, a web-based, collaborative review software was used for screening purposes (14, 15 Preprint). A similar protocol was followed by Liu et al. (16) in a meta-analysis and systematic review of treatments for posttraumatic stress disorder. The protocol of the current meta-analysis has been pre-registered at PROSPERO CRD42022339015 (17).

### Search databases & terms

The search was conducted on May 4, 2022. The search used 5 databases: Pubmed/Medline, PsycINFO, EMBASE, Web of Science, and CINAHL. The following terms were used: “ketamine”, “S-ketamine”, “esketamine”, “NMDA-receptor”, “military”, “combat”, “soldier”, “veteran”, “armed force”, “special force”, “air force”, “navy”, “army”, “marine” and “militia” (see **Supplementary Material** for string terms).

### Inclusion and exclusion criteria

Studies were included if they had a military (active duty or Veteran) population, administered ketamine by any route (e.g., oral, nasal, intravenous, intramuscular), and sought treatment for either of pain, depression, or PTSD. Studies were excluded if they were non-human (e.g., rodents), review (e.g., meta-analysis), gene/neuroscience (e.g., neurotransmission) studies, or mixed ketamine with other drugs (e.g., pain cream that combined ketamine along with other drugs).

### Study selection

At each screening level, two independent screeners reviewed each article. Interrater reliability (i.e., calculated as percent agreement) was high at both the title and abstract (92%) and full-text review (88%) stages. Conflicts were resolved at each stage as a team between the screeners and study leads (J.L., N.E., and J.G.) until consensus was reached.

### Data extraction

The following data was extracted from each study: study characteristics, study data, moderator information, and study rigour information. The study characteristics included: (1) *mean age* of participants, (2) *gender* (i.e., percent of men participants), and (3) *citizenship of participants* (e.g., American), and (4) type of treatment *protocol* implemented (depression or pain).

#### Meta-analysis

The study data included: (1) *study group* (i.e., ketamine or control^1^), (2) *sample size* between study groups, (3) *outcome* (i.e., pain or depression or PTSD), (4) *mental health category^2^* (e.g., depression, PTSD), and (5) *pre- and post-outcome mean and standard deviation*s.

The moderator information included: (1) *population targeted* (i.e., active duty or Veterans), (2) route of ketamine *administration* [i.e., oral, intravenous, intranasal, intramuscular, or not applicable (N/A)], (3) *duration* of ketamine therapy (in minutes e.g., 40 minutes), (4) *mean dose* of ketamine (e.g., 0.5 mg/kg), (5) *study design* [i.e., randomised controlled trial (RCT) - double blind, open label, or retrospective/chart review], (6) *concurrent treatment* (i.e., medication, psychological, both, none, or unknown), (7) whether the sample was *treatment resistant* (e.g., yes, no, unknown), (8) the *frequency* of ketamine administration (e.g., one time only use of ketamine), and (9) *chronicity* of the outcome ketamine was used to treat (i.e., acute, chronic, unknown).

Pre-outcome values were extracted by identifying the values of relevant outcome measures capturing the time period closest but prior to the first ketamine dose administration. Extraction of post-outcome values depended on certain elements of the study design. For studies that only administered ketamine once, outcome measure values taken at, or closest to 24 hours post-infusion were extracted. This procedure mimics that of Feder et al. (18), which selected 24 hours post-infusion as the primary study endpoint in order to allow time for any sedation or other side effects to resolve. Further, Feder et al. (18) noted a rapid reduction in psychological symptoms at this point, with lasting effects over multiple weeks. Given that the somatic effects from ketamine can usually be felt within 10 minutes and may persist up to three months (19), both psychological and somatic symptom changes can be captured at the 24 hours post infusion mark. For studies that administered multiple doses of ketamine over the study period, the first outcome measure value reported after the final infusion was extracted. In certain cases, including chart reviews or naturalistic study designs, the final outcome measures were taken prior to the final ketamine infusion. In these cases, we extracted the outcome measure values closest to the final ketamine infusion.

#### Systematic review

The study rigour information included: (1) whether *washout* of existing treatment(s) were implemented (i.e., yes, no, or unknown), (2) *dose range* of ketamine (e.g., 56 – 85 mg), (3) whether any *adverse effects* were noted (yes, no, or unknown), (4) *sample size* between groups, (5) *concurrent treatment*, (6) *study design*, and (7) whether the sample was *treatment resistant.*

To evaluate risk of bias among articles, the Mixed Method Appraisal Tool (MMAT; 20) was used. The MMAT is a validated assessment that can efficiently assess the quality of qualitative, quantitative, and mixed methods studies. All of the studies in this review were quantitative and were categorised as either quantitative randomised-control trial or quantitative non-randomised. They were then rated based on the corresponding MMAT criteria, relative to the type of study. Criteria included five specific appraisal questions that assess methodological characteristics (e.g., whether appropriate measurements were used) based on the study type. Criteria were extracted with ‘yes’, ‘no’, and ‘can’t tell’ as answer options.

### Data analysis

#### Meta-analysis

The meta-analysis was conducted using the Comprehensive Meta-Analysis (CMA) software version 3 (21). All information (study characteristics, study data, and moderator information) was inserted into CMA. Additionally, a correlation to account for between-subjects variance in within-subjects designs was required. A correlation of 0.77 was inserted (used in previous meta-analysis; 22). As well, effect direction was required to represent whether the change in outcomes were in line with our hypotheses (i.e., outcomes improved from pre- to post-ketamine; positive) or not (i.e., outcomes did not improve from pre- to post-ketamine; negative). Main analyses examined the effects of the ketamine group relative to the effects of the outcome measures. The effect size used was converted to Hedges’ *g* and interpreted based on Ferguson (23); 0.41 for a minimum effect representing a practically significant effect, 1.15 for a moderate effect, and 2.70 for a strong effect. Following the main analyses, moderator analyses were examined across outcomes. Moderator analyses were only conducted for sub-groups with a minimum of four samples (12). Given the low samples or lack of variability in citizenship, chronicity, and type of protocol, moderator analyses were not conducted but the data was instead used to characterise the sample of this meta-analysis. Following the main and moderator analyses, a continuous meta-regression was conducted using the moments method as the estimation framework to determine the influence of age and gender on the pooled effects of ketamine. Lastly, publication bias was examined using visual inspection of the funnel plot, Egger’s Regression test, Durval and Tweedie’s trim-and-fill, and classic fail-safe.

#### Systematic review

Studies were evaluated in categories to determine the rigour in which they were carried out. These categories included whether studies implemented a washout of existing treatment(s) prior to the commencement of ketamine trial, whether ketamine dose range was considered, whether researchers documented adverse effects, if sample sizes were sufficient, whether researchers allowed for concurrent treatments, the design of the overall study, and whether researchers included participants that were treatment resistant. To determine study rigour, we assigned each category a numerical value (see **Table 1**). After scoring each category, the scores were summed, and an overall rigour score was assigned. The following overall rigour scores were applied to each study: 0 to 4 (poor), 5 to 6 (moderate), and 7 to 10 (excellent).

**Table 1:** Study rigour scoring criteria.

Categories	Numerical Value		
	0	1	2
**Dose Range (by kg)**	Unknown	Fixed Dose	Variability Dose
**Adverse Effect**	Unknown	Yes *or* No	–
**Sample Size**	< 25 participants	≥ 25 participants	–
**Concurrent Treatment**	Unknown	Medication *and/or* Psychological	–
**Study Design**	Retrospective/Chart Review	Open Label	RCT (Double Blind)
**Treatment Resistant**	Unknown	Yes	–

RCT, Randomised Control Trial.

To assess the risk of bias of each study using the MMAT, we calculated the percentage of MMAT criteria met (i.e., the number of ‘yes’ responses). Hong et al. (20) does not suggest calculating an overall score; instead, studies were rated on the percentage of criteria met (i.e., 20, 40, 60, 80, 100; per 20).

## Results

### Meta-analysis

**Figure 1:**
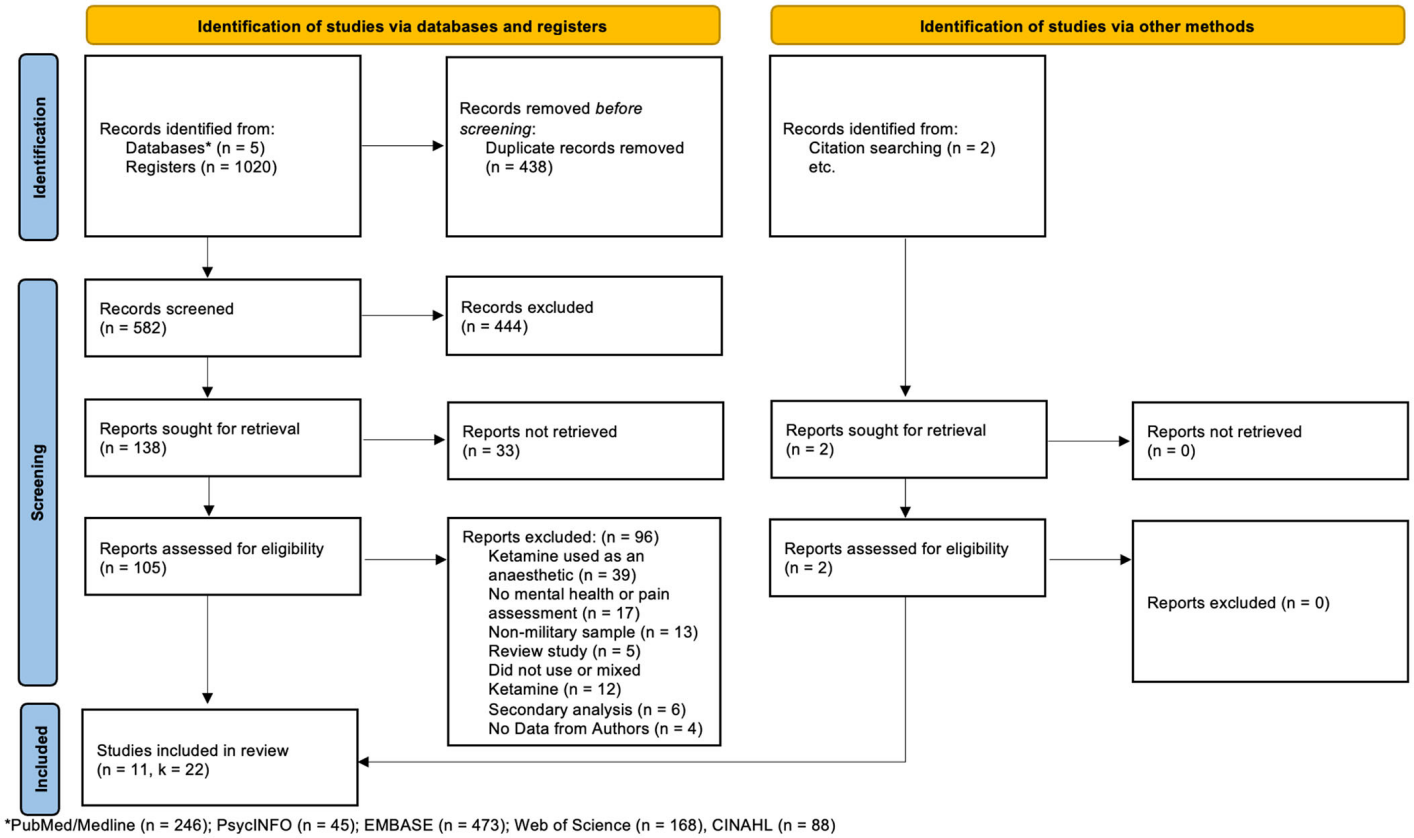
Preferred Reporting Items for Systematic Reviews (PRISMA) flow diagram (24).

The final samples included for this meta-analysis consisted of 11 articles with 22 independent samples (see **Figure 1**). Our meta-analysis included 384 military members that participated in a ketamine treatment group. Across samples, participants’ citizenship was mostly American (*k* = 20) with two samples consisting of Taiwanese participants (*k* = 2). Across samples, there were 8 studies adopting a depression-based protocol for use of ketamine, and 3 studies adopting a chronic-pain-based protocol for use of ketamine. Across all samples, ketamine was used to treat chronic ailments (see **Table 2**).

**Table 2:** Study data of included studies.

Author	Study Group	Protocol	Outcome	MH Category	N	Pre-Intervention		Post-Intervention	
						M	SD	M	SD
Albott et al. (29)	Ketamine	Depression	MH	PTSD	15	52.52	6.18	18.93	8.60
			MH	Depression	15	35.60	2.46	8.90	3.18
Artin et al. (30)	Ketamine	Depression	MH	PTSD	35	54.40	12.00	39.30	13.90
			MH	Depression	35	19.60	4.00	15.00	4.80
Bentley et al. (25)	Ketamine Group 1	Depression	MH	PTSD	15	58.72	12.17	54.28	17.17
			MH	Depression	15	21.79	2.90	19.47	4.30
	Ketamine Group 2	Depression	MH	PTSD	15	55.89	17.76	47.41	17.96
			MH	Depression	15	19.05	4.37	16.04	4.85
Chen et al. (31)	Ketamine Group 1	Depression	MH	Depression	24	23.04	4.85	13.25	7.08
	Ketamine Group 2		MH	Depression	23	23.09	4.80	15.26	7.98
Cohen et al. (32)	Ketamine	Pain	PN	–	9	5.10	1.80	0.70	1.00
Dadabayev et al. (26)	Ketamine Group 1	Pain	PN	–	10	25.43	3.08	18.79	2.86
			MH	PTSD	10	47.12	8.70	36.21	8.29
	Ketamine Group 2	Pain	PN	–	10	24.57	4.19	12.74	5.04
			MH	PTSD	10	18.40	3.85	10.38	3.94
Lijffijt et al. (33)	Ketamine Group 1	Depression	MH	Depression	11	32.55	5.64	18.72	4.21
	Ketamine Group 2		MH	Depression	5	35.80	2.42	23.53	6.70
	Ketamine Group 3		MH	Depression	4	35.50	2.05	22.37	3.28
Marton et al. (34)	Ketamine	Depression	MH	Depression	27	34.10	5.99	17.70	7.21
Pennybaker et al. (35)	Ketamine	Depression	MH	Depression	49	35.49	5.57	23.05	6.23
Polomano et al. (36)	Ketamine	Pain	PN	–	19	2.95	1.10	3.63	0.80
Shiroma et al. (37)	Ketamine	Depression	MH	Depression	13	29.90	2.30	7.00	2.30

MH, mental health; PN, pain; PTSD, posttraumatic stress disorder.

#### Overall analyses

*All Samples (k = 22):* To assess the effects of the ketamine intervention on all measures, we combined samples across all outcomes. A total of 22 samples were entered into a mixed, random effect model to determine the pooled effect of ketamine intervention on all outcome measures from pre- to post-intervention. The results indicated a significant difference across outcomes, *Q*(21) = 290.14, *p* <.001. The pooled effect size showed a positive moderate effect [*g* = 1.76, *SE* = 0.19, 95% *CI* (1.39, 2.13), *Z* = 9.26, *p* <.001]. Across all samples, effects were found to be considerably heterogeneous (*I*2 = 92.76), *Q*(21) = 290.14, *p* <.001. In comparing effects between depression and PTSD (k = 18) and pain (k = 4), results indicated no significant difference across outcomes, *Q*(1) = 0.00, *p* = .97.

#### Analyses by groups

*Depression and PTSD (k = 18):* To assess the effects of the ketamine intervention on depression and PTSD outcomes, we combined samples across studies. A total of 18 samples were entered into a mixed, random effect model to determine the pooled effect of ketamine intervention on mental health outcomes from pre- to post-intervention. The results indicated a significant difference across outcomes, *Q*(17) = 259.28, *p* <.001. The pooled effect size showed a positive moderate effect [*g* = 1.77, *SE* = 0.22, 95% *CI* (1.35, 2.19), *Z* = 8.26, *p* <.001].

*Pain (k = 4):* A total of 4 samples were entered into a mixed, random effect model to determine the pooled effect of ketamine intervention on the pain outcomes from pre- to postintervention. The results indicated a significant difference across outcomes, *Q*(3) = 30.80, *p* <.001. The pooled effect size showed a positive moderate effect [*g* = 1.75, *SE* = 0.51, 95% *CI* (0.76, 2.75), *Z* = 3.44, *p* = .001].

*Depression (k = 12) and PTSD (k = 6):* A total of 18 samples were entered into a mixed, random effects model to determine the pooled effect of depression outcomes relative to PTSD outcomes. Results indicated no significant difference across the type of mental health outcome, *Q*(1) = 2.73, *p* = .10. The pooled effect size showed a positive moderate effect for the depression outcomes [*g* = 2.06, *SE* = 0.29, 95% *CI* (1.50, 2.62), *Z* = 7.20, *p* <.001], and a positive moderate effect for the PTSD outcomes [*g* = 1.34, *SE* = 0.33, 95% *CI* (0.69, 1.98), *Z* = 4.05, *p* <.001]. Across all samples, effects were found to be considerably heterogeneous (*I*2 = 93.44), *Q*(17) = 259.28, *p* <.001.

#### Study outcomes across moderators

*Population targeted:* There was a significant difference in effect sizes based on the population targeted. While both active-duty and Veteran populations reported moderate effects, studies conducted with active-duty military personnel reported a slightly smaller effect relative to studies conducted with Veteran populations (see **Table 3** for test statistics; see **Supplementary Table 1** for moderator data of included studies).

**Table 3:** Test statistics for moderator analysis.

Moderators	*k*	*g*	*SE*	95% *CI*		*Z*	*Q*
				Lower	Upper		
**Population Targeted**							4.31*
Veteran	18	1.94^b^	0.24	0.48	2.40	8.24***	
Active Duty	4	1.21^b^	0.26	0.71	1.72	4.71***	
**Administration**							19.03***
Intravenous	18	2.10^b^	0.24	1.63	2.58	8.63***	
Internasal	4	0.74^a^	0.20	0.35	1.12	3.72***	
**Duration**							35.84***
40-minutes	14	2.49^b^	0.28	1.94	3.03	8.92***	
Unknown	7	0.67^a^	0.12	0.43	0.90	5.53***	
7-minutes	1	–	–	–	–	–	
**Mean Dose**							28.23***
0.5 mg/kg	11	2.75^c^	0.32	2.12	3.39	8.50***	
81 mg	4	0.74^a^	0.20	0.35	1.12	3.72***	
0.1 mg/kg	3	–	–	–	–	–	
0.2 mg/kg	2	–	–	–	–	–	
1 mg/kg	1	–	–	–	–	–	
**Study Design**							15.79*
RCT – Double	9	1.68^b^	0.19	1.30	2.06	8.75***	
Retrospective/Chart	7	0.97^a^	0.29	0.40	1.54	3.34**	
Open Label	6	3.61^c^	0.61	2.42	4.79	5.96***	
**Concurrent Treatment**							21.65***
Unknown	9	0.96^a^	0.18	0.62	1.31	5.48***	
Medication	7	3.55^c^	0.60	2.40	4.71	6.03***	
Both	4	1.79^b^	0.26	1.28	2.30	6.83***	
Psychological	2	–	–	–	–	–	
**Frequency**							24.50***
One Time	10	1.73^b^	0.19	1.37	2.10	9.29***	
6 Infusions	7	3.23^c^	0.58	2.10	4.36	5.61***	
8 Doses	4	0.74^a^	0.20	0.35	1.12	3.72***	
3 Infusions	1	–	–	–	–	–	
**Treatment Resistant**							0.18
Yes	16	1.82^b^	0.23	1.36	2.28	7.78***	
Unknown	6	1.65^b^	0.32	1.02	2.29	5.11***	

k, number of samples; g, Hedges’ g; SE, standard error; CI, confidence interval; Z, Fisher’s Z; Q, Q-statistics (Cochran’s observed dispersion); *p <.05; **p <.01; ***p <.001.

^a^ = minimum effect representing a practically significant effect.

^b^ = effect representing a moderate-sized effect.

c = effect representing a large-sized effect.

*Administration:* There was a significant difference in effect sizes based on the route of administration of ketamine. Studies administering ketamine intravenously yielded a moderate-sized effect while studies employing intranasal routes yielded a small effect.

*Duration:* There was a significant difference in effect sizes based on the duration of the ketamine exposure. In particular, studies that employed a 40-minute exposure yielded a moderate effect size.

*Mean dose:* There was a significant difference in effect sizes based on the mean dose of ketamine administered. Studies that reported a mean dose of 0.5 mg/kg yielded large effects while studies that reported a standard dose of 81 mg (regardless of weight) yielded small effects.

*Study design:* There was a significant difference in effect sizes based on the design of the study. Studies that are open labelled reported large sized effects. Randomised controlled trials (with double blinding) reported moderate sized effects. Retrospective and/or chart review studies reported small sized effects.

*Concurrent treatment:* There was a significant difference in effect sizes based on the allowance for, and type(s) of concurrent treatments permitted. Studies that allowed concurrent medication yielded a large effect, while studies that allowed for both psychological and pharmacological treatments (i.e., ketamine in addition to psychotherapy or other medication, or both) yielded a moderate effect.

*Frequency:* There was a significant difference in effect sizes based on the number of doses given. Studies that employed 6 infusions yielded a large effect. Studies that employed a one-time dose yielded a moderate effect. Studies that employed 8 doses yielded a small effect.

*Treatment resistance:* There were no significant differences in effect sizes based on whether study populations included individuals who are treatment resistant (as identified by the study authors). Studies that reported the inclusion of treatment-resistant populations and studies that did not report on their inclusion or exclusion both yielded moderate-sized effects with similar standard error and confidence intervals.

#### Meta-regression

**Figure 2:**
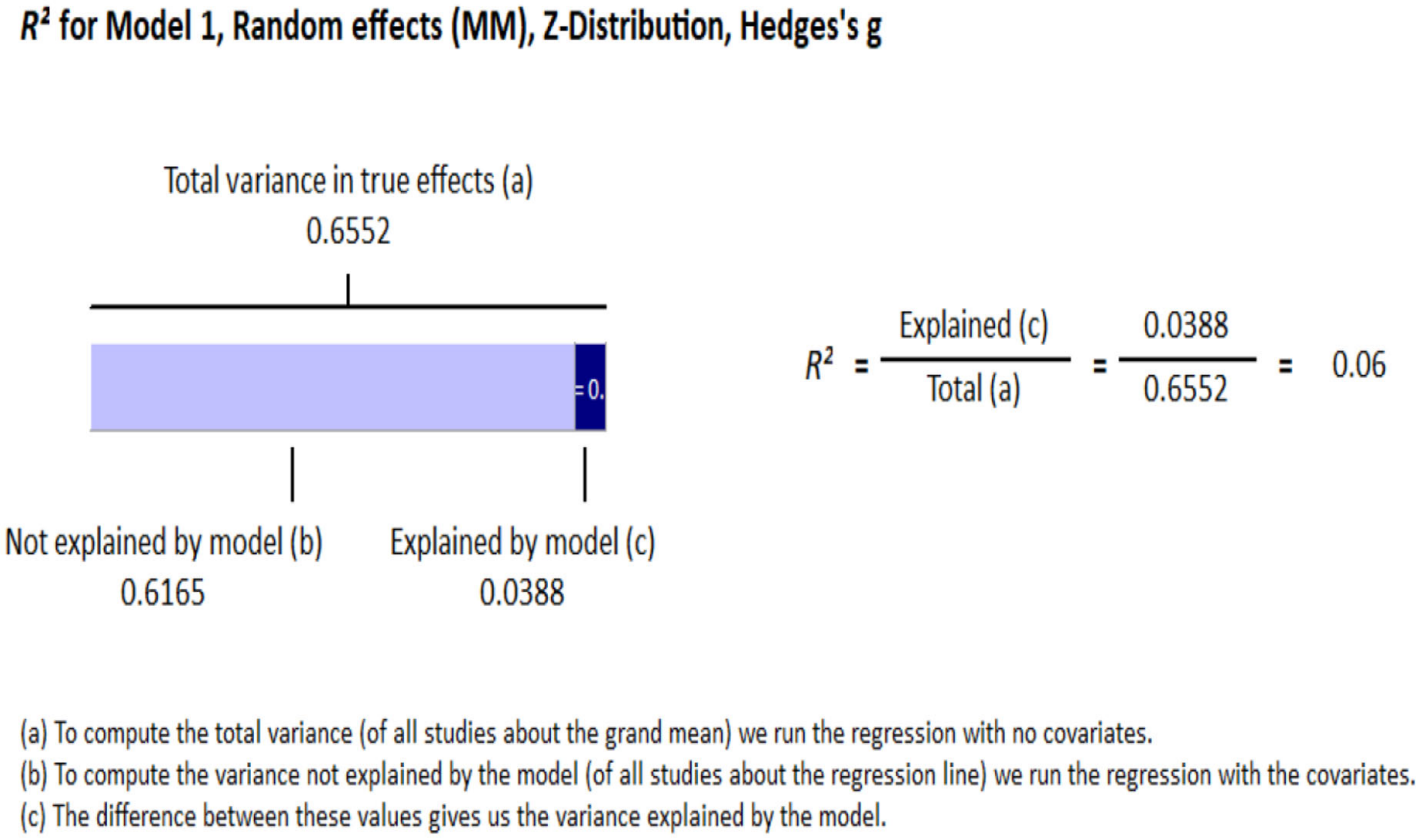
Meta-regression.

Age and gender were both entered as continuous variables, with gender coded a percentage of the sample that are men. The regression model significantly predicted explained variance within the data, *Q* = 15.68, *df* = 2, *p* <.001, accounting for 6% of the overall variance *adj R2* = .06 (see **Figure 2**). Specifically, ketamine is found to be more effective as age increases (*R2 change* = 0.02*; p* =.014), and in studies with a higher percentage of men (*R2 change* = 0.03*; p* =.002; see **Supplementary Table 1** for moderator data across included studies).

#### Publication bias

**Figure 3:**
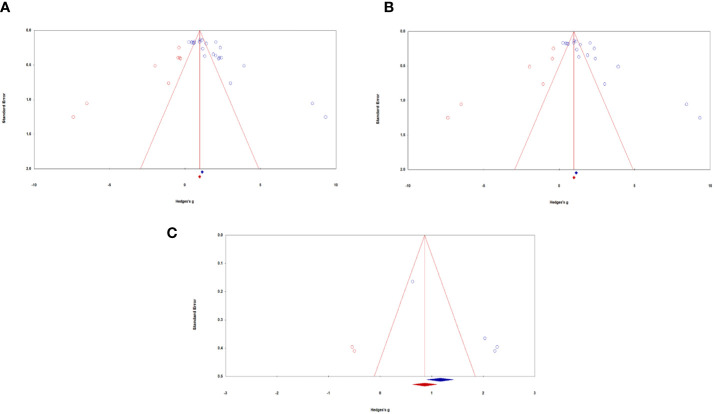
Funnel plots of standard error by Hedges’ *g* across samples: **(A)** All samples, **(B)** Mental health samples, **(C)** Pain samples. Blue circles represent actual studies, red circles represent imputed studies.

*All samples:* Visual inspection of the funnel plot indicated that the sampled studies were clustered towards the peak with wide scattering throughout. There were more studies scattered towards the right side compared with the left side of the funnel plot (see **Figure 3A**). Egger’s regression test detected a significant asymmetry, (*B*0) = 6.17, *t*(20) = 5.06, *p* <.001. For Durval and Tweedie’s (27) trim-and-fill, under the random-effects model, the point estimate and 95% confidence interval for the combined studies is 1.76 (1.38, 2.13). Using trim-and-fill, the imputed point estimate is 1.03 (0.68, 1.43). Classic fail-safe N revealed that it would take 4176 ‘null’ studies in order for the combined 2-tailed *p*-value to exceed .05. In other words, there would need to be 189.80 missing studies for every observed study for the effect to be nullified. Taken together, analyses suggest the presence of significant publication bias in the literature regarding ketamine administration for chronic pain, depression, and PTSD in military and Veteran populations. Results of the meta-analyses are thus encouraged to be cautiously interpreted with this in mind. In other words, true effects may be smaller than those reported, though it may be unlikely to affect the overall magnitude of categories of effects reported.

*Depression and PTSD:* Similar to all studies, visual inspection of the funnel plot indicated that the sampled studies were clustered towards the peak with wide scattering throughout. There were more studies scattered towards the right side compared with the left side of the funnel plot (see **Figure 3B**). Egger’s regression test detected a significant asymmetry, (*B*0) = 6.23, *t*(16) = 4.20, *p* <.001. For Durval and Tweedie’s (27) trim-and-fill, under the random-effects model, the point estimate and 95% confidence interval for the combined studies is 1.77 (1.35, 2.19). Using trim-and-fill, the imputed point estimate is 1.02 (0.56, 1.49). Classic fail-safe N revealed that it would take 2932 ‘null’ studies in order for the combined 2-tailed *p*-value to exceed .05. In other words, there would need to be 162.90 missing studies for every observed study for the effect to be nullified. Altogether, the presence of publication bias of depression and PTSD outcomes caution the careful interpretation of study results.

*Pain:* Visual inspection of the funnel plot indicated that the sampled studies were scattered towards the right side compared with the left side (see **Figure 3C**). Egger’s regression test detected a significant asymmetry, (*B*0) = 6.84, *t*(2) = 129.88, *p* <.01. For Durval and Tweedie’s (27) trim-and-fill, under the random-effects model, the point estimate and 95% confidence interval for the combined studies is 1.75 (0.75, 2.75). Using trim-and-fill, the imputed point estimate is 1.01 (0.10, 1.93). Classic fail-safe N revealed that it would take 106 ‘null’ studies in order for the combined 2-tailed *p*-value to exceed .05. In other words, there would need to be 26.5 missing studies for every observed study for the effect to be nullified. Taken together, analyses examining publication bias of pain outcomes suggest the presence of moderate publication bias. We thus encourage results to be interpreted with this in mind, as the true effect sizes may be slightly smaller than those reported in the current meta-analysis.

### Systematic review

This review included 11 articles; however, one article (25) was evaluated as two separate studies due to the differences in study designs between study samples resulting in a total of 12 studies being evaluated for study rigor. Except for one study, all others were evaluated to be low to moderate in rigour. The most rigorous category was the inclusion of treatment-resistant participants (i.e., treatment-resistant depression or PTSD or both). Of the 12 studies reviewed, 9 studies included treatment-resistant individuals, while 3 samples did not include this information. Next, the documentation of adverse effects and concurrent treatments were both reported in 8 studies, while 4 studies failed to document either. As for study design, most studies (5 out of 12) were retrospective/chart reviews, 4 were open-label studies, and 3 were randomised controlled trials. The least rigorous categories were sample size, and dose range. Using the minimum threshold for a small to medium effect, we determined that 9 out of the 12 studies did not meet the threshold of 25 participants (28). Further, 7 out of the 12 studies did not implement a range of ketamine dose administrations. Of the remaining 5 studies that did implement a variable dose, only 3 of the 5 considered dose by kilogram of the individuals. Only one study implemented a washout period to examine the independent effects of ketamine administration. Following data extraction, we did not assign a rigour rating to washout information given that a washout period is not specifically warranted unless a study employed a crossover design (to avoid carry-on effects). None of the included studies used a crossover design. As such, washout information is indicated descriptively in **Table 4**. We also tallied individual studies’ rigour scores to determine the overall rigour of the included samples. Of the included studies, 4 scored in the poor range, 7 in the moderate range, and only 1 scored in the excellent range (see **Table 4**).

**Table 4:** Raw data for study rigour.

Author	Dose Range	R	Adverse Effects	R	Sample Size	R	Concurrent Treatment	R	Study Design	R	Treatment Resistant	R	TR (/8)	TR
Albott et al (29)	Unkn	0	Yes	1	15	0	Medication	1	Open Label	1	Yes	1	4	Moderate
Artin et al (30)	28–84 mg	1	No	1	35	1	Unkn	0	Open Label	1	Yes	1	5	Moderate
Bentley et al. (25)^a^	56–85 mg	1	Unkn	0	15	0	Unkn	0	Retrospective/ Chart Review	0	Yes	1	2	Poor
Bentley et al. (25)^a^	0.5–1 mg/kg	2	Unkn	0	15	0	Unkn	0	Retrospective/ Chart Review	0	Yes	1	3	Poor
Chen et al (31)	Unkn	0	Unkn	0	24	0	Psychological	1	RCT - Double	2	Yes	1	4	Moderate
Cohen et al. (32)	Unkn	0	Yes	1	9	0	Medication	1	Open Label	1	Unkn	0	3	Poor
Dadabayev et al. (26)	Unkn	0	Yes	1	10	0	Both	1	RCT - Double	2	Unkn	0	4	Moderate
Lijffijt et al. (33)^W^	Unkn	0	Unkn	0	11	0	Unkn	0	RCT - Double	2	Yes	1	3	Poor
Marton et al. (34)	Unkn	0	No	1	27	1	Medication	1	Retrospective/ Chart Review	0	Yes	1	4	Moderate
Pennybaker et al. (35)	0.5–0.7 mg/kg	2	Yes	1	49	1	Medication	1	Retrospective/ Chart Review	0	Yes	1	6	Excellent
Polomano et al. (36)	0.06–0.12 mg/kg	2	Yes	1	19	0	Medication	1	Retrospective/ Chart Review	0	Unkn	0	4	Moderate
Shiroma et al. (37)	Unkn	0	Yes	1	13	0	Medication	1	Open Label	1	Yes	1	4	Moderate
Number of Rigor Ratings by Category														
Total # of 0	**7**		4		**9**		4		**5**		3			
Total # of 1	2		**8**		3		**8**		4		**9**			
Total # of 2	3		–		–		–		3		–			

^a^, Bentley et al. (25) was assessed as two studies due to the variations in study design between groups; ^W^, implemented a treatment washout period; R, rigor rating numerical values per category; TR, total rigor rating score; Unkn, unknown; TR score ranges: 0 to 3 (poor), 4 to 5 (moderate), and 6 to 8 (excellent); bolded text indicates the highest number of ratings in each category.

### Risk of bias (Mixed Method Appraisal Tool)

The methodological quality of the included studies (*N* = 11) varied: 45% of the articles met ≤60% of MMAT criteria [5/11; 40% (*n* = 2), 60% (*n* = 3)], while 55% of the articles met >60% of MMAT criteria [6/11; 80% (*n* =5), 100% (*n* = 1)]. Notably, the most common criterion was a quality threshold of 80% (see **Supplementary Material** for MMAT scores of all included articles).

## Discussion

This meta-analysis and systematic review served as a first step towards comprehensive evaluations of the effectiveness of ketamine for depression, PTSD, and pain outcomes in military populations. Averaging across studies, improvements in symptoms were evident, with the use of ketamine leading to significant reductions. These effects were of moderate-to-large effect, with minor influences associated with older age and more men in study. In addition, the reductions in symptoms were observed in both active-duty and Veteran populations, and across different routes of ketamine administration, frequencies of ketamine administration, duration of ketamine treatments, dosage, study design, and allowances for concurrent treatments. Overall, assessment of the quality of the evidence was adequate, with most studies meeting the majority of the criteria measured by the MMAT. However, while the results of the MMAT indicate that the findings from this review are robust, findings should be cautiously interpreted given the low sample size, novelty of the research topic, low study rigour, and evidence of publication bias. Together, this review provides some evidence establishing ketamine’s promise as a potential option for the treatment of depression, PTSD, and chronic pain in military populations.

### Comparability to other populations

Although the reported effects of ketamine treatments in military samples were largely convergent with reviews conducted with civilian populations (see 6, 38), there are some nuanced distinctions in effects across mental health outcomes. Specifically, in our sample, we found a moderate-to-large effect for depression (*g* = 2.06), which was slightly larger than those found in the Price et al. (39) civilian samples for depression (*r* ≤ 0.29). Several explanations may account for these distinctions. First, it may be that the symptom prevalence and severity experienced by military populations are greater than that of civilian populations. Although there is a dearth of research examining the comparability of symptoms, ample research has identified higher rates of mental illness, as well as higher mental health service use among Veterans compared to civilians (40–42). Further, based on the nature of their work, it may be expected that the traumatic encounters as a result of training, deployment, and other occupational stress injuries within military populations may contribute to more chronic, persistent forms of mental illnesses. Indeed, research has found that up to 1 in 7 Veterans may have a form of treatment-resistant depression (43). In our meta-analytic sample, a large majority of the samples contained Veterans and individuals considered ‘treatment-resistant’ (to depression, PTSD, or both), both of which likely demonstrated chronic PTSD symptoms. In comparison, a review of ketamine for early incidence of PTSD for active-duty soldiers suggested it may be ineffective (44). Taken together, the allowance for both inclusion of treatment-resistant populations, as well as concurrent treatments may indicate that ketamine may be particularly useful for chronic PTSD samples. It may be the case that the addition of ketamine could contribute to improved symptoms overall while existing treatments work to stabilise symptoms.

### Factors that influence ketamine effectiveness

In examining factors that may differentially influence effect sizes, some differences emerge. Specifically, the degree of symptom reduction was slightly larger in Veteran populations compared to active duty. However, it should be noted that both groups were found to have moderate effect sizes, and thus these differences may be explained in several ways. First, the larger degree of change in symptomatology may be attributed to Veteran groups experiencing more severe symptoms, with thereby more room for improvement. Indeed, examinations of the baseline levels of pain and mental health outcomes found Veterans to have significantly higher scores compared to active-duty samples. In addition, participation in ketamine trials and/or use of ketamine may be stigmatised or perceived to be of higher risk for active-duty members of the military compared to Veterans (45). Indeed, the US Department of Veterans’ Affairs imposes strict limits on ketamine treatments for depression, which may contribute to the perceived and practical barriers of seeking treatments (46).

With regards to the delivery of ketamine, intravenous administration, with a mean dose of 0.5mg/kg and delivered over 6 infusions appears to be most effective relative to 8 doses. However, this protocol of delivery is specific to mental health protocols of administering ketamine to treat depression (47). It should also be noted that all studies which used 6 infusions were delivered intravenously while all studies which used 8 doses were delivered intranasally. Therefore, it may be likely that the effect size observed between frequency of administration map on to the observed differences between routes of administration found in this review.

Lastly, with respect to designs of studies, several features emerged as influential in contributing to larger reported effects. These include open label studies, with allowances for concurrent medication treatment of participants. These characteristics may contribute to stronger effect sizes for several reasons. First, the experimental nature of ketamine may invite internally motivated treatment-seekers, who may have found traditional treatment approaches ineffective. Indeed, our evidence suggests that the inclusion of treatment-resistant samples did not influence effect sizes, such that the effects were similar. Second, with regards to the effects of ketamine, it is difficult to have a placebo condition, nor is the potential to be placed in placebo desirable for participants who are participating in the hopes of benefitting from the effects of ketamine. As such, it is important to consider that open-label trials may inherently contain biases in a way that accentuates the benefits of ketamine therapy.

In this review, studies which allowed for concurrent treatments in the form of both psychotherapies and pharmacotherapies only included Veteran populations (see **Supplementary Table 2**). It may be more likely that Veterans have tried various treatments before, have comorbid conditions, and/or needed these treatments in order to stabilise symptoms. Lastly, our review also highlights the tolerability, feasibility, and acceptability of ketamine when used concurrently with existing treatments, which may be attractive to military and Veteran populations often experiencing comorbidities and receiving multiple treatments from different providers. Taken together, findings from this review suggest that the effects of ketamine may be robust for treatment-resistant populations, and that motivation of the treatment seeker may play a key role in its experienced and observed effects.

### Limitations and future directions in research

An important consideration regarding factors that may contribute to differences in reported effect sizes may be differences in protocol. Specifically, the factors that led to larger effect sizes (i.e., intravenous administration, mean dosage, frequency of dosage, and duration) were typical of the mental health protocol of administering ketamine to treat depression (47). The observed larger effects as a result of any of these characteristics may be confounded by the larger proportion of samples adopting this protocol in this review. First, it is unknown whether mental health protocols for ketamine contribute to similar reductions in pain, and vice versa for pain on depression and PTSD outcomes. Secondly, it is also unclear whether the entire protocol, or elements within the protocol, such as the mean dose, frequency, and duration of the dosage may differentially drive the observed effects. Indeed, results from the various moderator analyses included in this meta-analysis may indicate that some moderating variables confound with others (e.g., the effects observed with respect to frequency are likely tied to the route of administration). Various ketamine-assisted psychotherapy protocols have been used to treat a wide range of mental health disorders with significant variability in intervention structure and study design (48); however, there is limited research that examines this specific protocol in Veteran populations. It is thus important to identify and distinguish the contributing factors of effect sizes found. For example, ketamine for pain protocols includes elements that may reduce symptoms of mental health conditions, such as supportive therapy and breathing exercises (49). The conscious experience of ketamine by the patient may also be crucial to experiencing therapeutic benefits (see 50). Future research exploring these differences may wish to consider distinguishing components that delineate differential or similar responses in military populations seeking care.

Another important limitation is that most of the included studies did not simultaneously assess for both mental health and pain outcomes, with the exception of Dadabayev et al. (26), which evaluated the effects of ketamine administration on both pain and PTSD. This may contribute to potential biases in expected outcomes based on measured versus actual outcomes not measured. As such, the true effects of ketamine studies for military populations may be partially undermined by the missing information, lack of available information, and low study rigour. Distinguishing which components contribute to ketamine’s mechanism of action, and for which protocol (mental health or pain) may be of particular importance in advancing this literature. In addition, robust research is needed to systematically examine the differential adaptations of these protocols across different settings and over time. For example, it may be important to distinguish the effects of ketamine with respect to the contexts under which ketamine is delivered (e.g., supportive environments, supportive therapies accompanying, individual clinicians administering). We thus strongly urge future studies to prioritise the examination and documentation of variabilities in protocols of deliveries before consolidating consensus in standards of delivery for ketamine.

## Conclusion

The current study evaluated a growing body of evidence on the effectiveness of ketamine in reducing symptoms of pain, depression, and PTSD in military samples. As military and Veteran populations display a higher prevalence and chronicity of pain and mental health conditions, exploring the viability of alternative treatment options is essential. This is especially true for individuals who may be considered treatment-resistant, and/or have exhausted conventional forms of treatment, or have found them to be ineffective. Evidence evaluating the rigour of ketamine studies conducted with military samples underscores the need to conduct additional rigorous and empirical studies with an emphasis on sample size, variability in dosing, and chronicity and durability of observed effects. While ketamine offers an avenue for alternative therapy given its potential for improvements, future research should also aim to evaluate the overall risks vs benefits when determining appropriateness of use.

## Author contributions

JL: Writing – review & editing, Writing – original draft, Visualization, Supervision, Project administration, Methodology, Funding acquisition, Formal analysis, Data curation, Conceptualization. NE: Writing – review & editing, Writing – original draft, Visualization, Supervision, Project administration, Methodology, Investigation, Data curation, Conceptualization. JG: Writing – review & editing, Writing – original draft, Methodology, Formal Analysis, Data curation. CB: Writing – review & editing, Data curation. RP: Writing – review & editing, Methodology. SW: Writing – review & editing, Conceptualization. AB: Writing – review & editing, Conceptualization. BL: Writing – review & editing, Conceptualization. EA: Writing – review & editing, Conceptualization. TW: Writing – review & editing. AN: Writing – review & editing, Supervision, Resources, Project administration, Methodology, Conceptualization. DR: Writing – review & editing, Supervision, Resources, Project administration, Conceptualization.

## Footnotes

1 Data for only two control groups (one active, one placebo) was available and were not included in this review.

2 Data for only PTSD and depression mental health outcomes were available within the final included samples and were therefore the only mental health outcomes examined in this review.
