# Supplementary material for: Ketamine in the effective management of chronic pain, depression, and posttraumatic stress disorder for Veterans: A meta-analysis and systematic review
Source: Front Psychiatry. 2024 Jun 24;15:1338581. doi: 10.3389/fpsyt.2024.1338581 (PMC11228764; doi:10.3389/fpsyt.2024.1338581)
Supplement: Supplementary file 1 [file DataSheet_1.docx]

**Supplementary Material**

**Pubmed/Medline**

1. (Ketamine or S-ketamine or esketamine or NMDA-receptor).mp. [mp=title, abstract,

original title, name of substance word, subject heading word, floating subheading word,

keyword heading word, organism supplementary concept word, protocol supplementary

concept word, rare disease supplementary concept word, unique identifier, synonyms]

2. military or combat or soldier* or veteran* or armed force* or special force* or air force*

or navy or army or marine* or militia*).mp. [mp=title, abstract, original title, name of

substance word, subject heading word, floating subheading word, keyword heading word,

organism supplementary concept word, protocol supplementary concept word, rare

disease supplementary concept word, unique identifier, synonyms]

3. 1 and 2

**PsycINFO**

1. (Ketamine or S-ketamine or esketamine or NMDA-receptor).mp. [mp=title, abstract,

heading word, table of contents, key concepts, original title, tests & measures, mesh

word]

2. (military or combat or soldier* or veteran* or armed force* or special force* or air force*

or navy or army or marine* or militia*).mp. [mp=title, abstract, heading word, table of

contents, key concepts, original title, tests & measures, mesh word]

3. 1 and 2

**EMBASE**

1. (Ketamine or S-ketamine or esketamine or NMDA-receptor).mp. [mp=title, abstract,

heading word, table of contents, key concepts, original title, tests & measures, mesh

word]

2. (military or combat or soldier* or veteran* or armed force* or special force* or air force*

or navy or army or marine* or militia*).mp. [mp=title, abstract, heading word, table of

contents, key concepts, original title, tests & measures, mesh word]

3. 1 and 2

**Web of Science**

(Ketamine or S-ketamine or esketamine or NMDA-receptor) AND (military or combat or soldier

or veteran or "armed force" or "special force" or "air force" or navy or army or marine or militia)

[abstract]

**CINAHL**

(Ketamine or S-ketamine or esketamine or NMDA-receptor) AND (military or combat or soldier

or veteran or "armed force" or "special force" or "air force" or navy or army or marine or militia)

[abstract]

**Table S1.** Moderator Data of Included Studies

| **Author** | **Pop. Targeted** | **Mean Age** | **Males (%)** | **Admin.** | **Duration** | **Mean Dose** | **Study Design** | **Concurrent Treatment** | **Frequency** | **Tx Resistant** |
| --- | --- | --- | --- | --- | --- | --- | --- | --- | --- | --- |
| Albott et al  (2018) – 2 samples of data | Veterans | 52 | 67 | Intravenous | 40 minutes | 0.5 mg/kg | Open Label | Medication | 6 Infusions | Yes |
| Artin et al  (2022) – 2 samples of data | Veterans | 45 | 71 | Intranasal | Unkn | 81 mg | Open Label | Unkn | 8 Doses | Yes |
| Bentley et al (2022) – 4 samples of data | Veterans | 49 | 53 | Intranasal | Unkn | 81 mg | Retrospective/  Chart Review | Unkn | 8 Doses | Yes |
|  | Veterans | 49 | 53 | Intravenous | Unkn | 1 mg/kg | Retrospective/  Chart Review | Unkn | 6 Infusions | Yes |
| Chen et al  (2018)^a –^ 2 samples of data | Active Duty | 49 | 13 | Intravenous | 40 minutes | 0.5 mg/kg | RCT - Double | Psychological | One Time | Yes |
|  | Active Duty | 45 | 26 | Intravenous | 40 minutes | 0.2 mg/kg | RCT - Double | Psychological | One Time | Yes |
| Cohen et al (2004) – 1 sample | Active Duty | 46 | 40 | Intravenous | 7 minutes | 0.1 mg/kg | Open Label | Medication | One Time | N/A |
| Dadabayev et al (2020) – 4 samples of data | Veterans | 45 | 64 | Intravenous | 40 minutes | 0.5 mg/kg | RCT - Double | Both | One Time | N/A |
|  | Veterans | 44 | 90 | Intravenous | 40 minutes | 0.5 mg/kg | RCT - Double | Both | One Time | N/A |
| Lijffijt et al (2022) – 3 samples of data | Veterans | 61 | 73 | Intravenous | 40 minutes | 0.5 mg/kg | RCT - Double | Unkn | One Time | Yes |
|  | Veterans | 62 | 40 | Intravenous | 40 minutes | 0.2 mg/kg | RCT - Double | Unkn | One Time | Yes |
|  | Veterans | 67 | 100 | Intravenous | 40 minutes | 0.1 mg/kg | RCT - Double | Unkn | One Time | Yes |
| Marton et al (2019) – 1 sample | Veterans | 54 | 63 | Intravenous | 40 minutes | 0.5 mg/kg | Retrospective/  Chart Review | Medication | 6 Infusions | Yes |
| Pennybaker et al (2021) – 1 sample | Veterans | 53 | 75 | Intravenous | 40 minutes | 0.5 mg/kg | Retrospective/  Chart Review | Medication | 6 Infusions | Yes |
| Polomano et al (2013) – 1 sample | Active Duty | 25 | 100 | Intravenous | Unkn | 0.1 mg/kg | Retrospective/  Chart Review | Medication | 3 Infusions | N/A |
| Shiroma et al (2014) – 1 sample | Veterans | 52 | 100 | Intravenous | 40 minutes | 0.5 mg/kg | Open Label | Medication | 6 Infusions | Yes |

*Notes*. Admin. = route of ketamine administration; Tx = treatment; Unkn = unknown; RCT = randomized control trial; Both = medication and psychological; ^a^ = all studies included American participants except Chen et al (2018) which included Taiwanese participants. All studies treated chronic ailments.

**Table S2**. Samples across Moderator Characteristics

| **Moderators** | ***k*** | **Population Targeted** | | **Administration** | | **Study Design** | | |
| --- | --- | --- | --- | --- | --- | --- | --- | --- |
|  |  | Veteran | Active Duty | Intravenous | Internasal | RCT – Double Blind | Retrospective / Chart | Open Label |
| **Population Targeted** |  |  |  |  |  |  |  |  |
| Veteran | 18 | -- | -- |  |  |  |  |  |
| Active Duty | 4 | -- | -- |  |  |  |  |  |
| **Administration** |  |  |  |  |  |  |  |  |
| Intravenous | 18 | 14 | 4 | -- | -- |  |  |  |
| Internasal | 4 | 4 | 0 | -- | -- |  |  |  |
| **Study Design** |  |  |  |  |  |  |  |  |
| RCT – Double Blind | 9 | 7 | 2 | 9 | 0 | -- | -- | -- |
| Retrospective / Chart | 7 | 6 | 1 | 5 | 2 | -- | -- | -- |
| Open Label | 6 | 5 | 1 | 4 | 2 | -- | -- | -- |
| **Duration** |  |  |  |  |  |  |  |  |
| 40-minutes | 14 | 12 | 2 | 14 | 0 | 9 | 2 | 3 |
| **Mean Dose** |  |  |  |  |  |  |  |  |
| 0.5 mg/kg | 11 | 10 | 1 | 11 | 0 | 6 | 2 | 3 |
| 81 mg | 4 | 4 | 0 | 0 | 4 | 0 | 2 | 2 |
| **Concurrent Treatment** |  |  |  |  |  |  |  |  |
| Medication | 7 | 5 | 2 | 7 | 0 | 0 | 3 | 4 |
| Both | 4 | 4 | 0 | 4 | 0 | 4 | 0 | 0 |
| **Frequency** |  |  |  |  |  |  |  |  |
| One Time | 10 | 7 | 3 | 10 | 0 | 9 | 0 | 1 |
| 6 Infusions | 7 | 7 | 0 | 7 | 0 | 0 | 4 | 3 |
| 8 Doses | 4 | 4 | 0 | 0 | 4 | 0 | 2 | 2 |

*Notes*. RCT = randomized control trials

**Table S3.** Mixed Methods Appraisal Tool

| **Author** | **S1** | **S2** | **2.1** | **2.2** | **2.3** | **2.4** | **2.5** | **3.1** | **3.2** | **3.3** | **3.4** | **3.5** | **Total** |
| --- | --- | --- | --- | --- | --- | --- | --- | --- | --- | --- | --- | --- | --- |
| Albott et al  (2018) | Yes | Yes |  |  |  |  |  | Yes | Yes | No | No | Yes | 60% quality criteria met |
| Artin et al  (2022) | Yes | Yes |  |  |  |  |  | Yes | Yes | Yes | Yes | Yes | 100% quality criteria met |
| Bentley et al (2022) | Yes | Yes |  |  |  |  |  | Yes | Yes | Yes | No | Yes | 80% quality criteria met |
| Chen et al  (2018) | Yes | Yes | CT | Yes | Yes | Yes | Yes |  |  |  |  |  | 80% quality criteria met |
| Cohen et al (2004) | Yes | Yes |  |  |  |  |  | Yes | No | Yes | Yes | Yes | 80% quality criteria met |
| Dadabayev et al (2020) | Yes | Yes | CT | No | Yes | Yes | Yes |  |  |  |  |  | 60% quality criteria met |
| Lijffijt et al (2022) | Yes | Yes | Yes | No | CT | Yes | No |  |  |  |  |  | 40% quality criteria met |
| Marton et al (2019) | Yes | Yes |  |  |  |  |  | No | Yes | Yes | No | CT | 40% quality criteria met |
| Pennybaker et al (2021) | Yes | Yes |  |  |  |  |  | Yes | Yes | No | Yes | Yes | 80% quality criteria met |
| Polomano et al (2013) | Yes | Yes |  |  |  |  |  | No | Yes | Yes | Yes | Yes | 80% quality criteria met |
| Shiroma et al (2014) | Yes | Yes |  |  |  |  |  | No | No | Yes | Yes | Yes | 60% quality criteria met |

*Notes*. CT = can’t tell; for more information see Hong et al., 2018.

**Reference List of Included Articles**

Albott, C. S., Lim, K. O., Forbes, M. K., Erbes, C., Tye, S. J., Grabowski, J. G., Thuras, P., Batres-y-Carr, T. M., Wels, J., and Shiroma, P. R. (2018). Efficacy, safety, and durability of repeated ketamine infusions for comorbid posttraumatic stress disorder and treatment-resistant depression. *J. Clin. Psychiatry*. 79:3. <https://doi.org/10.4088/JCP.17m11634>

Artin, H., Bentley, S., Mehaffey, E., Liu, F. X., Sojourner, K., Bismark, A. W., Printz, D., Lee, E. E., Martis, B., De Peralta, S., Baker, D. G., Mishra, J., and Ramanathan, D. (2022). Effects of intranasal (S)-ketamine on Veterans with co-morbid treatment-resistant depression and PTSD: A retrospective case series. *EClinicalMedicine*. *48*:101439. <https://doi.org/10.1016/j.eclinm.2022.101439>

Bentley, S., Artin, H., Mehaffey, E., Liu, F., Sojourner, K., Bismark, A., Printz, D., Lee, E. E., Martis, B., De Peralta, S., Baker, D. G., Mishra, J., and Ramanathan, D. (2022). Response to intravenous racemic ketamine after switch from intranasal (S)‐ketamine on symptoms of treatment‐resistant depression and post‐traumatic stress disorder in Veterans: A retrospective case series. *Pharmacotherapy: The Journal of Human Pharmacology and Drug Therapy*. 42:3, 272–279. <https://doi.org/10.1002/phar.2664>

Chen, M.-H., Li, C.-T., Lin, W.-C., Hong, C.-J., Tu, P.-C., Bai, Y.-M., Cheng, C.-M., and Su, T.-P. (2018). Cognitive function of patients with treatment-resistant depression after a single low dose of ketamine infusion. *J. Affect. Disord*. 241, 1–7. <https://doi.org/10.1016/j.jad.2018.07.033>

Cohen, S. P., Chang, A. S., Larkin, T., and Mao, J. (2004). The intravenous ketamine test: A predictive response tool for oral dextromethorphan treatment in neuropathic pain. *Anesth. Analg.*1753–1759. <https://doi.org/10.1213/01.ANE.0000136953.11583.7B>

Dadabayev, A. R., Joshi, S. A., Reda, M. H., Lake, T., Hausman, M. S., Domino, E., & Liberzon, I. (2020). Low dose ketamine infusion for comorbid posttraumatic stress disorder and chronic pain: A randomized double-blind clinical trial. *Chronic Stress*. 4, 247054702098167. <https://doi.org/10.1177/2470547020981670>

Lijffijt, M., Murphy, N., Iqbal, S., Green, C. E., Iqbal, T., Chang, L. C., Haile, C. N., Hirsch, L. C., Ramakrishnan, N., Fall, D. A., Swann, A. C., Al Jurdi, R. K., and Mathew, S. J. (2022). Identification of an optimal dose of intravenous ketamine for late-life treatment-resistant depression: A Bayesian adaptive randomization trial. *Neuropsychopharmacology*. 47:5, 1088–1095. <https://doi.org/10.1038/s41386-021-01242-9>

Marton, T., Barnes, D. E., Wallace, A., and Woolley, J. D. (2019). Concurrent use of buprenorphine, methadone, or naltrexone does not inhibit ketamine’s antidepressant activity. *Biol. Psychiatry*. 85:12, e75–e76. <https://doi.org/10.1016/j.biopsych.2019.02.008>

Pennybaker, S., Roach, B. J., Fryer, S. L., Badathala, A., Wallace, A. W., Mathalon, D. H., and Marton, T. F. (2021). Age affects temporal response, but not durability, to serial ketamine infusions for treatment refractory depression. *Psychopharmacology*. 238:11, 3229–3237. <https://doi.org/10.1007/s00213-021-05939-z>

Polomano, R. C., Buckenmaier, C. C., Kwon, K. H., Hanlon, A. L., Rupprecht, C., Goldberg, C., and Gallagher, R. M. (2013). Effects of low-dose iv ketamine on peripheral and central pain from major limb injuries sustained in combat. *Pain Med*. 14:7, 1088–1100. <https://doi.org/10.1111/pme.12094>

Shiroma, P. R., Albott, C. S., Johns, B., Thuras, P., Wels, J., & Lim, K. O. (2014). Neurocognitive performance and serial intravenous subanesthetic ketamine in treatment-resistant depression. *Int. J. Neuropsychopharmacol.* 17:11, 1805–1813. <https://doi.org/10.1017/S1461145714001011>
